# Effects of bacterial and viral pathogen-associated molecular patterns (PAMPs) on multidrug resistance (MDR) transporters in brain endothelial cells of the developing human blood–brain barrier

**DOI:** 10.1186/s12987-023-00409-4

**Published:** 2023-01-31

**Authors:** Phetcharawan Lye, Enrrico Bloise, Stephen G. Matthews

**Affiliations:** 1 Department of Physiology, Temerty Faculty of Medicine, University of Toronto, Medical Sciences Building Room 3207, 1 King’s College Circle, Toronto, ON M5S 1A8 Canada; 2 Lunenfeld-Tanenbaum Research Institute, Sinai Health System, Toronto, ON Canada; 3 Departamento de Morfologia, Instituto de Ciências Biológicas, N3-292, Universidade Federal de Minas Gerais, Belo Horizonte, Minas Gerais 31270-901 Brazil; 4 Department of Obstetrics & Gynaecology, University of Toronto, Toronto, Canada; 5 Department of Medicine, Temerty Faculty of Medicine, University of Toronto, Toronto, ON Canada

**Keywords:** Blood–brain barrier (BBB), Toll like receptor (TLR), Lipopolysaccharide (LPS), Polyinosinic polycytidylic acid (Poly I:C), Single-stranded RNA (ssRNA), Multidrug resistance (MDR) transporters, P-glycoprotein (P-gp/*ABCB1*), Breast cancer resistance protein (BCRP/*ABCG2*)

## Abstract

**Background:**

The multidrug resistance (MDR) transporters, P-glycoprotein (P-gp, encoded by *ABCB1*) and breast cancer resistance protein (BCRP/*ABCG2*) contribute to the blood–brain barrier (BBB), protecting the brain from drug exposure. The impact of infection on MDR in the developing human BBB remains to be determined. We hypothesized that exposure to bacterial and viral pathogen-associated molecular patterns (PAMPs) modify MDR expression and activity in human fetal brain endothelial cells (hfBECs) isolated from early and mid-gestation brain microvessels**.**

**Methods:**

We modelled infection (4 h and 24 h) using the bacterial PAMP, lipopolysaccharide (LPS; a toll-like receptor [TLR]-4 ligand) or the viral PAMPs, polyinosinic polycytidylic acid (Poly I:C; TLR-3 ligand) and single-stranded RNA (ssRNA; TLR-7/8 ligand). mRNA expression was assessed by qPCR, whereas protein expression was assessed by Western blot or immunofluorescence. P-gp and BCRP activity was evaluated by Calcein-AM and Chlorin-6 assays.

**Results:**

TLRs-3,4 and 8 were expressed by the isolated hfBECs. Infection mimics induced specific pro-inflammatory responses as well as changes in P-gp/*ABCB1* or BCRP/*ABCG2* expression (P < 0.05). LPS and ssRNA significantly decreased P-gp activity at 4 and 24 h in early and mid-gestation (P < 0.03-P < 0.001), but significantly increased BCRP activity in hfBECs in a dose-dependent pattern (P < 0.05-P < 0.002). In contrast, Poly-IC significantly decreased P-gp activity after 4 h in early (P < 0.01) and mid gestation (P < 0.04), but not 24 h, and had no overall effect on BCRP activity, though BCRP activity was increased with the highest dose at 24 h in mid-gestation (P < 0.05).

**Conclusions:**

Infectious PAMPs significantly modify the expression and function of MDR transporters in hfBECs, though effects are PAMP-, time- and dose-specific. In conclusion, bacterial and viral infections during pregnancy likely have profound effects on exposure of the fetal brain to physiological and pharmacological substrates of P-gp and BCRP, potentially leading to altered trajectories of fetal brain development.

**Supplementary Information:**

The online version contains supplementary material available at 10.1186/s12987-023-00409-4.

## Introduction

The blood–brain barrier (BBB) actively modulates transport of factors from the peripheral circulation into the central nervous system (CNS) [1]. The BBB is formed by endothelial cells within brain capillaries, ensheathed externally by pericytes and astrocyte-foot processes, and maintained through the formation of tight junction between adjacent endothelial cells [2–4]. This endothelial barrier also includes the multidrug-resistance (MDR) transporters, P-glycoprotein (P-gp; encoded by *ABCB1*) and breast cancer resistance protein (BCRP; *ABCG2*) [4]. These members of the ATP-binding cassette (ABC) superfamily of efflux transporters prevent exposure of the brain to a large range of molecules, including specific cytokines, chemokines, xenobiotics, environmental toxins, steroids and waste products—that may be present in the peripheral circulation [4–7]. MDR transporters are enriched at the luminal surface of the plasma membrane of brain capillary endothelial cells, and their activity not only protects the CNS from the entry of neurotoxins but can also limit access to a range of therapeutic drugs as well as an array of physiological factors into the brain parenchyma [4, 8].

Functional expression of P-gp and BCRP has been demonstrated in isolated human fetal brain endothelial cells (hfBECs) derived from early and mid-gestation [9]. These results indicate that P-gp and BCRP at the BBB protect the developing CNS from early-gestation and throughout mid-pregnancy. However, there is limited information about how co-morbidities or common challenges during pregnancy, such as maternal malnutrition, stress/synthetic glucocorticoid exposure and infection disrupt the protective brain barrier provided by these MDR transporters.

P-gp and BCRP are involved in neuroinflammatory responses within the CNS [10] and can be modified by infection and inflammation in different biological barriers, including the BBB [4, 11–13], as well as the intestine [14], the placenta [15–20] and the yolk sac [21–23]. Previously, we showed that exposure to bacterial pathogen-associated molecular patterns (PAMPs), modeled through lipopolysaccharide exposure (LPS; highly enriched in Gram-bacteria and a toll-like receptor [TLR]-4 ligand) alters the activity and expression of these drug-transporters in adult human cerebral microvascular endothelial (hCMEC/D3) cells [11]. Similarly, exposure of hCMEC/D3 cells to viral PAMPs such as the double-stranded viral RNA (dsRNA) antigen polyinosinic:polycytidylic acid (Poly I:C; TLR-3 ligand) and the single-stranded viral RNA (ssRNA) antigen (TLR-7/8 ligand), altered the expression and function P-gp/*ABCB1* and BCRP/*ABCG2* [11]. Alterations in MDR expression and activity in the BBB by infective mimics (modelled by bacterial and viral PAMPs) may lead to altered accumulation of several compounds in the brain parenchyma. Given the vulnerable state of the CNS during in utero development, increased exposure of the developing brain to MDR substrates including therapeutic drugs, toxicants and inflammatory cytokines [4], may alter the normal trajectory of brain development and lead to poor behavioral and cognitive outcomes.

A number of bacterial and viral infective agents have been associated with multiple adverse pregnancy outcomes, including infection-mediated preterm birth, chorioamnionitis and fetal CNS inflammation/maldevelopment [24, 25]. Infection during pregnancy with some strains of Gram-negative bacteria including *Escherichia coli*, *Ureaplasma urealyticum* and *Mycoplasma hominis,* primarily resulting from bacterial vaginosis, may induce infective preterm labour [20, 25, 26], and adversely impact fetal brain development and subsequent neurobehavioral function [27]. This may occur through promotion of host systemic- or neuroinflammatory responses that increase the permeability of the brain microvasculature and favor entry of microorganisms into the developing brain [4, 28–31]. Similarly, viral infection during pregnancy increases risk of preterm labor, and may induce mild to severe fetal CNS abnormalities [32, 33]. Specifically, intrauterine exposure to parvovirus, cytomegalovirus (CMV), varicella-zoster (VZV), rubella and Zika virus (ZIKV) infections amongst others, have been shown to promote fetal neurologic infections or promote potent cytokine/chemokine responses capable of eliciting white matter injury during development [27]. However, whether these infective agents alter MDR expression and function at the developing human BBB, through direct activation of specific TLRs in early and mid-pregnancies is unknown. Since in vitro and in vivo studies using immortalized adult human BECs and BECs derived from various animals models have demonstrated that infective PAMPs alter the activity of P-gp and or BCRP in a PAMP-dependent manner [11, 34], we hypothesize that bacterial and/or viral PAMPs modify the expression and / or function of P-gp and BCRP within hfBECs, and disrupt the protection they provide to the developing brain—potentially altering the levels of harmful substrates within the developing CNS and contributing to fetal brain damage induced by intrauterine infection. Therefore, we investigated the impact of bacterial and viral PAMPs, modeling activation of TLRs 3, 4 and 7/8 on P-gp and BCRP activity in hfBECs, to determine whether different infective pathways modify the protective barrier function afforded by P-gp and BCRP in the developing CNS.

## Methods

### Ethical approval

Early and mid-gestation fetal brains were collected following elective termination of pregnancy, as previously described [9]; in order to detect possible gestational-age dependent patterns of MDR responses to infection. Early-gestation fetal brains (N = 6) were obtained at 11.3–13.5 weeks of gestation, whereas second-trimester fetal brains (N = 6) were derived from 17.3–18 weeks of gestation. Fetal brains were collected by the Women’s and Infants’ Health BioBank program at Sinai Health System through written informed consent (protocol #18–0057-E) obtained in adherence to the policies of the Sinai Health System and the University of Toronto Research Ethics Boards, which do not allow the collection of any identifying or clinical information from elective pregnancy terminations.

### Isolation of human brain endothelial cells

Early and mid-gestation human fetal brain endothelial cells (hfBECs) were isolated as previously described [9]. In brief, after isolation from fetal brains, cells were plated on type I rat tail collagen- (50 μg/mL; 5056, Advanced BioMatrix, San Diego, CA, USA) coated tissue culture flasks (353136, ThermoFisher scientific, Mississauga, ON, CA) and grown in a 37 °C/5% CO_2_-incubator in EndoGROTM-MV Complete Culture Media Kit®, (SCME004, Millipore, Blvd, ON, Canada), supplemented with recombinant human epidermal growth factor (5 ng/mL), L-Glutamine (10 mM), hydrocortisone hemisuccinate (1.0 µg/mL), heparin sulfate (0.75 U/mL), ascorbic acid (50 µg/mL), 20% FBS, penicillin (100 IU/mL), streptomycin (100 IU/mL) (15,140–122, Life Technologies, Carlsbad, California, USA), 1% normocin antibiotic (ant-nr-2, Invivogen, San Diego, CA, USA) at 20% O_2_ (5% CO_2_, 37 °C). hfBECs were used at passage 4 in all subsequent experiments, and subjected to PAMP treatments as described below.

### Exposure of human fetal brain endothelial cells (hfBECs) to PAMPs

Early and mid-gestation hfBECs were plated in 96-well plates (6,000 cells/well) for activity studies, or in 6-well plates (200,000 cells/well) for expression analysis and were grown to confluency, cultured for 24 h at 20% O_2_ (5% CO_2_, 37 °C) in EndoGROTM-MV Complete Culture Media (as described above). For P-gp and BCRP activity studies, EndoGRO media was then replaced with Dulbecco's Modified Eagle Medium (DMEM) (21063029, Thermo Fisher Scientific) and was supplemented with 10% charcoal-stripped (CS)-FBS (Wisent, Saint-Jean-Baptiste, QC, CA) for 24 h. hfBECs were then exposed for either 4 h or 24 h (to investigate a possible time-dependent response) to, LPS (L4391, Sigma-Aldrich, St. Louis, Missouri, USA), to Poly I:C (P9582, Sigma-Aldrich) or their vehicle (water); to simian virus 40 large T antigen (SV40) ssRNA (lrna40, Invivogen, San Diego, California, USA), or vehicle/lyovec (lyec-12, Invivogen) at 0.001 to 1 μg/mL, in order to determine PAMP-dose responses in hfBECs, in dose ranges previously shown to elicit an inflammatory response in other cell types [11, 35–37]. All LPS treatments were performed using aliquots prepared from the same original vial. Similarly, all Poly I:C treatments were performed using aliquots prepared from the same original vial. Due to small vial size, ssRNA treatments were performed using different vials but from same batch. Cultures were then subjected to activity analysis as described below. For P-gp/*ABCB1* and BCRP/*ABCG2* protein and mRNA analysis, hfBECs were plated (6-well plate) and cultured (as described above) and exposed to LPS (0.01 μg/mL), Poly I:C (1.0 μg/mL), ssRNA (0.001 μg/mL), or vehicle/lyovec for 24 h. These doses were selected as they had significant effects on P-gp and BCRP activity, after which they were collected and stored at − 80 °C for subsequent analysis. In the primary experiment, for logistical reasons, assessment of the effects of PAMPs on mRNA, protein and transport function were undertaken in two separate batches (early and mid-gestation).

### Immunofluorescence

Early and mid-gestation hfBECs (N = 3 subjects in duplicates/group) were cultured to approximately 75% confluence specifically for immunofluorescence analysis as described previously [38]. In brief, hfBECs were rinsed with cold PBS, fixed with 4% paraformaldehyde (Electron Microscopy Sciences) for 15 min and then permeabilized with 0.05% Tween 20 in PBS (5 min, room temperature). Autofluorescence was reduced using 0.1% Sudan Black in 70% ethanol (1 min) and non‐specific binding was blocked using 2% BSA for 1 h. Slides with hfBECs were incubated with primary antibodies, TLR-3 (ab62566, 1:500, Abcam, Toronto, ON, Canada), TLR-4 (ab22048, 1;200, Abcam), TLR-8 (ab180610, 1:200, Abcam), von Willebrand Factor (vWF) (ab11713,1:500, Abcam), anti-rabbit IgG (ab171870, 1:500, Abcam) and anti-mouse IgG2b (X0944D,1:500, Dako, Burloak, ON, Canada). overnight at 4 °C. Slides were then incubated with fluorescent secondary antibodies; the anti-mouse Alexa 488 (A21202, 1:1000), the anti-rabbit Alexa 488 (A21206, 1:1000), the anti-rabbit Alexa 568 (A10042, 1:1000), or the anti-sheep Alexa 555 (A21436, 1:000) secondary antibodies (Thermo Fisher Scientific) and counterstained with DAPI (1 μg/mL, 1 h). Fluorescent microscopy was performed using a spinning disc confocal microscope at various magnification (Leica DMI6000 B, Concord, ON, Canada).

### Immunoblotting

Western blot analysis was performed as previously described [39, 40]. Briefly, protein isolated from cultured cells (N = 6/group, one well per treatment/subject (6-well plate)) was extracted by sonication using lysis buffer (1 mol/L Tris–HCL pH 6.8, 2% SDS, 10% glycerol) which included a protease and phosphatase inhibitor cocktail (78,420, Thermo Scientific). The protein concentration was determined with the Pierce BCA Protein Assay kit (Thermo Scientific). Proteins were separated by electrophoresis (20 μg 100 V, 1 h) using SDS polyacrylamide gels (8% or 12%). Proteins were then transferred (10 min) to polyvinylidene fluoride (PVDF) membrane using Trans-Blot Turbo (Bio-Rad). Membranes were blocked with skim milk (5%; 1 h, room temperature). The primary antibodies used were P-gp (ab170903, dilution 1:1,000; Abcam), BCRP (ab108312, dilution 1:1,000; Abcam) and the loading control used was ERK2 (sc-1647, dilution 1:2,000; Santa Cruz Biotechnology). Blots were incubated with primary antibodies overnight (4 °C). The PVDF membranes were subsequently incubated with HRP-linked anti-rabbit secondary antibody (1:10,000, 1 h; GE Healthcare Bio-Science, Baie d’Urfe, QC, Canada). Protein-antibody complexes were detected by incubating the PVDF membranes with Laminate Crescendo Western HRP Substrate (5 min; Millipore) and chemiluminescence was detected under UV using the ChemiDoc™ MP Imaging system (Bio-Rad). The protein band intensity was quantified using Image Lab™ software.

### Quantitative real time PCR (qPCR)

Total RNA was isolated from cultured hfBECs (N = 6/group; one well per treatment/subject (6-well plate)) using the RNeasy Plus Universal Mini Kit (73404, Qiagen, Toronto, ON, Canada), as previously described [41]. RNA concentration and purity were assessed using a NanoDrop1000 Spectrophotometer (Thermo Scientific). RNA was reverse transcribed into cDNA using the iScript Reverse Transcription Supermix (Bio-Rad). *ABCB1*, *ABCG2*, interleukin (*IL-6)*, *IL-8*, interferon (*IFN)α***,** C–C motif chemokine ligand 2 *(CCL2)* also known as monocyte chemoattractant protein 1 (MCP-1), *TLR-3, TLR-4*, and *TLR-8* mRNA levels were measured (in triplicate per subject) by qPCR using SYBR Green reagent (Sigma-Aldrich) and the CFX 380 Real-Time system C1000 TM Thermal Cycler (Bio-Rad), with the following cycling conditions: initial denaturation at 95 °C (2 min) followed by 39 cycles of denaturation at 95 °C (5 s) and combined annealing and extension at 60 °C (20 s). Gene expression was normalized to the geometric mean of DNA topoisomerase 1 (*TOP1*), the zeta polypeptide (*YWHAZ*) and succinate ubiquinone oxidoreductase (*SDHA*) which exhibited stable expression after LPS, Poly I:C, and ssRNA treatment. The developmental profile of TLR expression was normalized to the geometric mean of DNA topoisomerase 1 (*TOP1*) and the zeta polypeptide (*YWHAZ*). The primer sequences of all the assessed genes are shown in Table 1.

**Table 1:** List of primers used in this study

Gene name	Sequence	References
*ABCB1*	Forward: 5’GCCCTTGTTAGACAGCCTCA-3’	[38]
	Reverse: 5’GGCTTTGTCCAGGGCTTCTT-3’	
*ABCG2*	Forward: 5'-TGGAATCCAGAACAGAGCTGGGGT-3'	[38]
	Reverse: 5'-AGAGTTCCACGGCTGAAACACTGC-3'	
*IL-6*	Forward: 5'-TGCAGAAAAAGGCAAAGAAT-3'	[34]
	Reverse: 5'-CTGACCAGAAGAAGGAATGC-3'	
*IL-8*	Forward: 5'-TGGGAACAAGAGGGCATCTG-3'	[36]
	Reverse: 5'-CCACCACTGCATCAAATTCATG-3'	
*IFN-α*	Forward: 5'-GGAGGTTGTCAG AGCAGA AA-3'	[35]
	Reverse: 5'-CAGGGGTGAGAGTCTTTG AA-3'	
*TLR-3*	Forward: 5'-TTACGAAGAGGCTGGAATGG-3'	[36]
	Reverse: 5'-AGGAACTCCTTTGCCTTGGT -3'	
*TLR-4*	Forward: 5'-ATTTGTCTCCACAGCCACCA-3'	[36]
	Reverse: 5'-ACAGGAAACCCCATCCAGAG-3'	
*TLR-8*	Forward: 5'-TCTTACGGATCCGCTGCCGTAGCC-3’	[77]
	Reverse: 5'-TCCTGGGGATCCAAGAGGGAAGAG-3’	
*SDHA*	Forward: 5'-TGGGAACAAGAGGGCATCTG-3'	
	Reverse: 5'-CCACCACTGCATCAAATTCATG-3'	
*YWHAZ*	Forward: 5'-CCGCCAGGACAAACCAGTAT-3'	[62]
	Reverse: 5'-CAC ATC ACA GCT CCC CAC CA-3'	
*TOP1*	Forward: 5'-GATGAACCTGAAGATGATGGC-3'	[51]
	Reverse: 5'-TCAGCATCATCCTCATCTCG-3'	

### P-gp, BCRP and esterase activity assays

P-gp function was assessed as described previously [9, 11, 12] with adaptations. Briefly, hfBECs (N = 6/group, each treatment/subject run in technical triplicates; i.e. three wells per donor were seeded and treatments and activity measures were undertaken on all three wells in a single experiment) were seeded and treated as described above. Cells were washed twice with warm Tyrode salts’ solution (T2145, Sigma) supplemented with sodium bicarbonate (1 g/L; S6014, Sigma). Cells were incubated with P-gp substrate calcein-acetoxymethyl ester (Ca-AM, 177831, 10^−6^ M, Sigma; 37 °C, 5% CO_2_, 1 h). After incubation with Ca-AM, the plates were placed on ice and the cells were washed twice with ice-cold Tyrode salts’ solution (Sigma), followed by cell lysis with 1% Triton X-100 (X100, Sigma) lysis buffer. The cellular content of Ca-AM was measured with a fluorescent microplate reader at excitation and emission wavelengths of 485 nm and 510 nm.

Esterase activity was also assessed, as described previously [9, 12]. Ca-AM is a non-fluorescing P-gp substrate, which is actively converted to fluorescent calcein by esterase enzymes in the cell. In cells that express P-gp, Ca-AM is transported out of the cell before this conversion [42]. To confirm that esterase activity was not affected by LPS (0.01 ug/ml), Poly I:C (0.01 ug/ml) and ssRNA (0.001 ug/ml), hfBECs were treated for 24 h. Cells were washed before the addition of a warm lysis buffer containing 10^−6^ M calcein-AM. Conversion of calcein-AM to calcein, following treatment, was assessed immediately after 1 h of incubation with lysis buffer, as described above.

BCRP function was assessed as previously described [9, 11] with adaptations. Briefly, hfBECs were seeded and treated with bacterial and viral infection as described above. The BCRP substrate Chlorin e6 (Ce6; 2 uM; SC-263067, Santa Cruz Biotechnology) was pre-incubated with Tyrode salts’ solution (Sigma) in a 37 °C bead bath for 30 min. Ce6 solution was then loaded into hfBECs (37 °C, 5% CO_2_, 1 h). After incubation with Ce6, the plates were placed on ice and the cells were washed twice with ice-cold Tyrode salts’ solution (Sigma), followed by cell lysis with 1% Triton X-100 (X100, Sigma) lysis buffer. The cellular content of Ce6 was measured with a fluorescent microplate reader at excitation and emission wavelengths of 407 nm and 667 nm.

### Statistical analyses

Analysis was undertaken to determine the effects of 3 different PAMPs (LPS, Poly I:C and ssRNA) in early- and in mid-pregnancy hfBECs. Data analyses were performed with Prism version 8 (GraphPad Software Inc., San Diego, CA, USA). The Grubb’s method was used to identify outliers (which were removed at the subject level), and the D’Agostino–Pearson test assessed normality of distribution. For P-gp and BCRP activity assays, triplicates of each subject were averaged, and a mean of the vehicle treatment calculated. For each PAMP-treatment, values were divided by their respective mean vehicle value to create a relative measure to control. These data were then used for statistical analysis. Differences among groups were compared using the non-parametric Friedman test, followed by Dunn’s multiple comparisons test comparing all doses against the respective control group (vehicle). Differences in protein and mRNA levels following treatment with TLR ligands were assessed by paired t-test. Differences were considered statistically significant when *p* < 0.05. All data are presented as mean ± S.D. Importantly, analysis of mRNA, protein, and transport activity in early and in mid-gestation groups, were designed and run in two separate batches (due to experimental and analytic limitations). This prevented the opportunity to statistically compare potential gestational-age effects using a Two-way ANOVA approach, though patterns of response within groups could be compared.

## Results

### Bacterial and viral PAMPs decrease P-gp activity in hfBECs

**Fig. 1:**
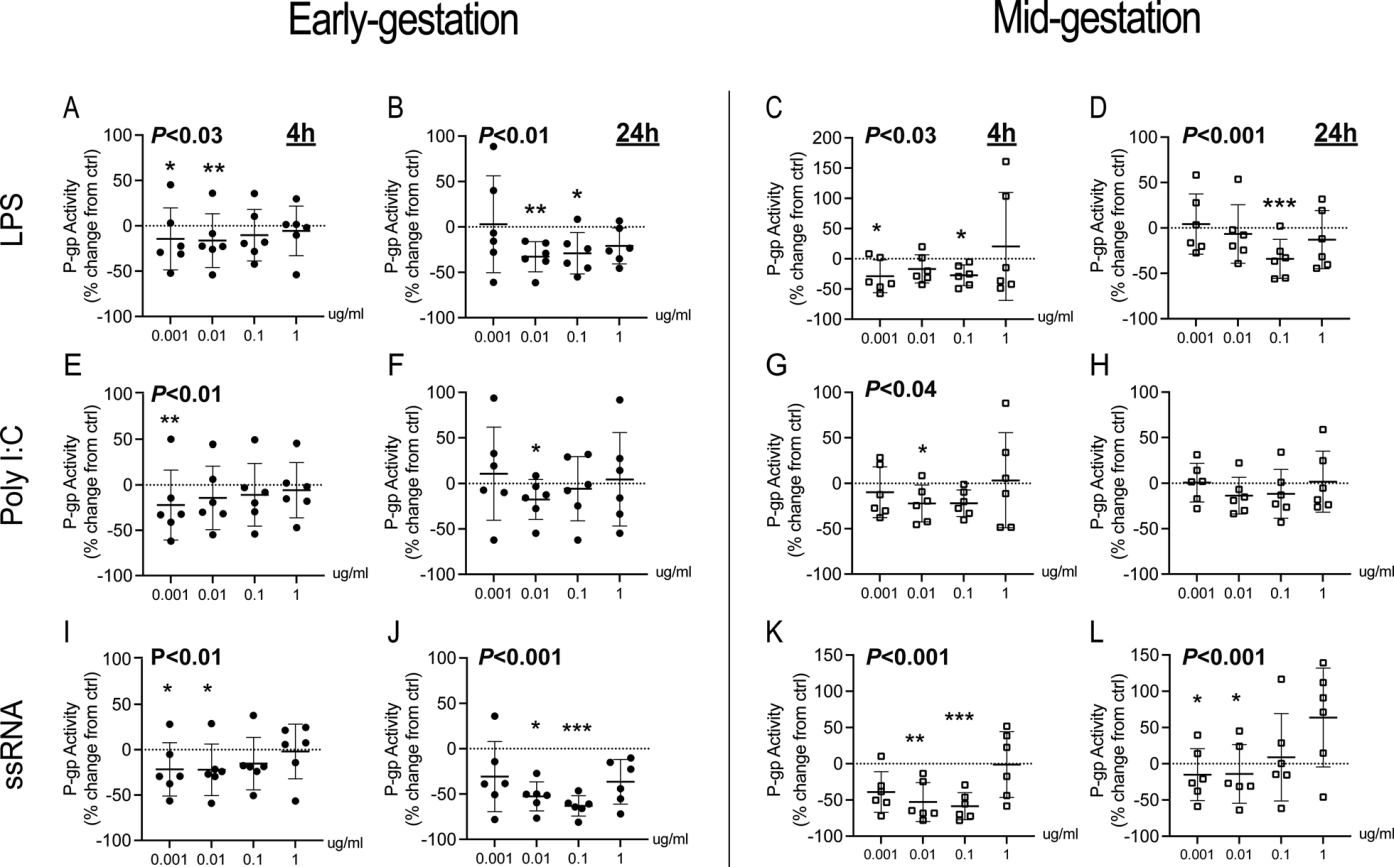
Bacterial and viral PAMPs decreased P-gp activity in early and mid-gestation human primary fetal brain endothelial cells (hfBECs), in a time and dose-specific manner. Percent change in P-gp activity in hfBECs following treatment with LPS (**A–D**), Poly I:C (**E–H**) or ssRNA (**I–L**) compared to control (water for LPS and Poly I:C; Lyovec for ssRNA) at 4 h and 24 h. P-gp activity is displayed as percent mean ± SD change from control. N = 6/group. Statistical differences were tested using Friedman test, followed by Dunn’s multiple comparisons test comparing all doses against the respective control group. Overall effects of treatment, within each age and timepoint, are presented at the top of each graph. Specific dose-effects are indicated as follows; *p < 0.05, **p < 0.01, ***p < 0.001

To simulate the effects of bacterial and viral infection on P-gp activity in early and mid-gestation, hfBECs were exposed to LPS, Poly I:C and ssRNA (0.001–1 μg/mL) for 4 h or 24 h. The Friedman test determined overall treatment effects at each timepoint, and Dunn’s multiple comparisons test determined specific dose-effects. In early-gestation, overall LPS led to a significant decrease in P-gp activity at 4 h (P < 0.03) and 24 h (P < 0.01 Fig. 1A, B). There were significant specific decreases (p < 0.05-*p* < 0.01) in P-gp activity after 4 h and 24 h at doses (0.001–0.1 μg/mL). In mid-gestation, overall LPS led to a significant decrease in P-gp activity at 4 h (P < 0.03) and 24 h (P < 0.001; Fig. 1C, D). Specifically, LPS (0.001 μg/mL and 0.1 μg/mL) resulted in a significant decrease (*p* < 0.05) in activity at 4 h, and at 0.1 ug/mL (p < 0.001) after 24 h (Fig. 1C, D). In early gestation, overall Poly I:C led to a significant decrease in P-gp activity at 4 h (P < 0.01; Fig. 1E, F). At specific doses, Poly I:C (0.001 μg/mL) decreased (*p* < 0.01) P-gp activity after 4 h exposure, while Poly I:C (0.01 μg/mL) decreased P-gp activity at 24 h (*p* < 0.05). In mid-gestation, overall Poly I:C led to a significant decrease in P-gp activity at 4 h (P < 0.04). At specific doses, Poly I:C (0.01 μg/mL) decreased (*p* < 0.05) P-gp activity after 4 h exposure, but had no effect at 24 h (Fig. 1G, H). In early gestation, overall ssRNA led to a significant decrease in P-gp activity at 4 h (P < 0.01) and 24 h (P < 0.001; Fig.  1I-J). At specific doses, ssRNA treatment significantly decreased P-gp activity at 4 h (*p* < 0.05; 0.001 and 0.01 μg/mL) and 24 h (0.01 and 0.1 μg/mL). In hfBECs derived at mid-gestation, overall ssRNA led to a significant decrease in P-gp activity at 4 h (P < 0.001) and 24 h (P < 0.001; Fig. 1K, L). At specific doses, ssRNA treatment significantly decreased P-gp activity at 4 h (0.01 and 0.1 μg/mL; P < 0.01) and 24 h (0.001 and 0.01 μg/mL; P < 0.05). Esterase activity was not affected by exposure to LPS (0.01 ug/mL), Poly I:C (0.01 ug/mL) or ssRNA (0.001 ug/mL) for 24 h (Additional file 1: Fig.S1).

### Bacterial and viral PAMPs increase BCRP activity in hfBECs

**Fig. 2:**
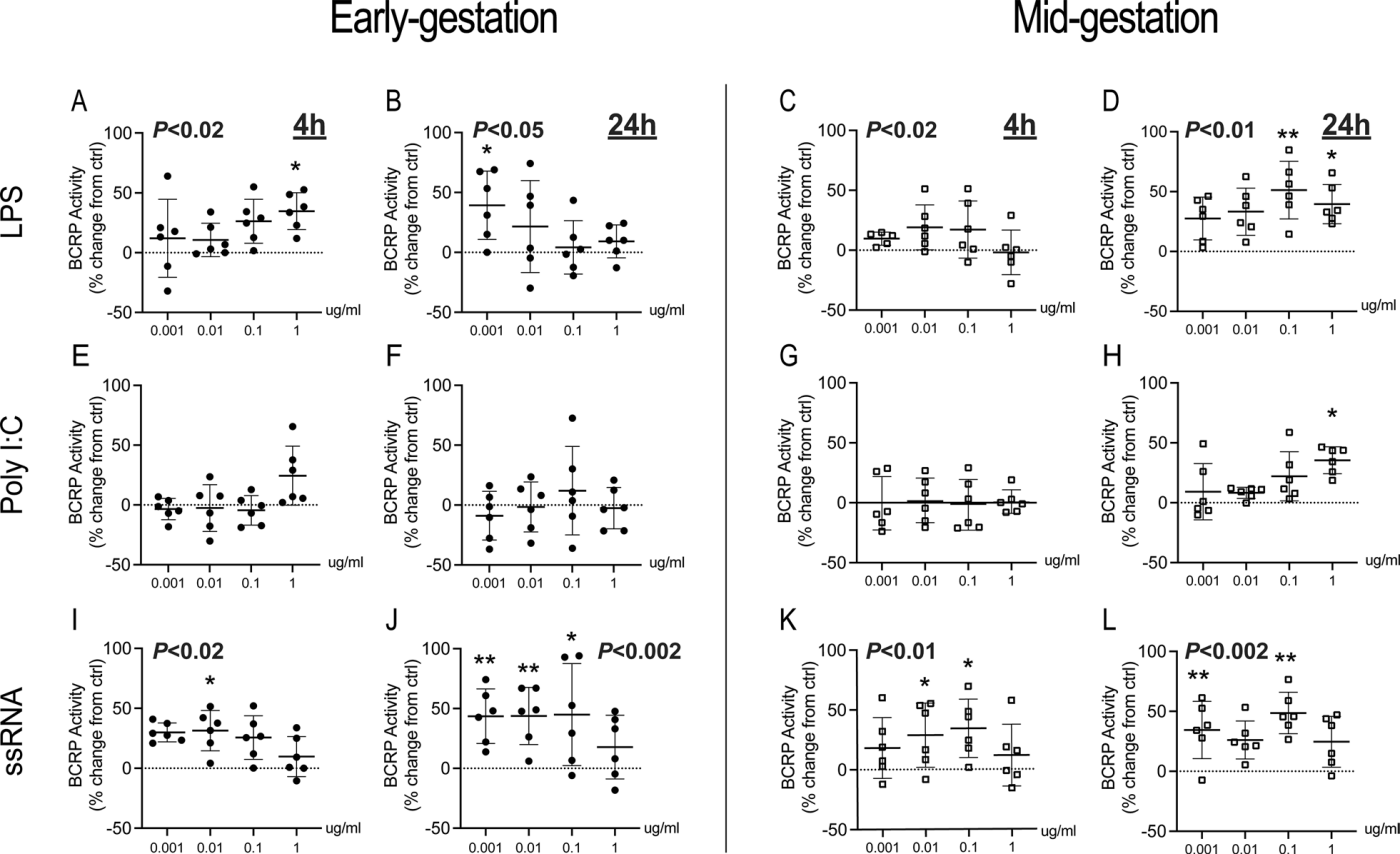
Bacterial and viral PAMPs increased BCRP activity in early and mid-gestation human primary fetal brain endothelial cells (hfBECs), in a time- and dose-specific manner. Percent change in BCRP activity in hfBECs following treatment with LPS (**A–D**), Poly (I:C) (**E–H**) or ssRNA (**I–L**) compared to control (water for LPS and Poly I:C; Lyovec for ssRNA) at 4 h and 24 h. BCRP activity is displayed as percent mean ± SD change from control. N = 6/group (if N < 6, an outlier has been removed). Statistical differences were tested using Friedman test, followed by Dunn’s multiple comparisons test comparing all doses against the respective control group. Overall effects of treatment, within each age and timepoint, are presented at the top of each graph. Specific dose-effects are indicated as follows; *p < 0.05, **p < 0.01

To probe the effects of bacterial and viral infection on BCRP activity in early and mid-gestation, hfBECs were exposed to LPS, Poly I:C and ssRNA. In early-gestation, overall LPS led to a significant increase in BCRP activity at 4 h (P < 0.02) and 24 h (P < 0.05; Fig. 2A, B). At specific doses, LPS increased BCRP activity (*p* < 0.05) after 4 h of exposure at a high dose (1 μg/mL), and after 24 h at the lowest dose (0.001 μg/mL). In mid-gestation, overall LPS significantly increased in BCRP activity at 4 h (P < 0.02) and 24 h (P < 0.01). While there were no dose-specific effects at 4 h, the two highest doses of LPS treatment significantly increased BCRP activity at the 24 h time-point (Fig. 2C, D). There were no overall effects of Poly I:C on BCRP activity (Fig. 2E–H). However, Poly I:C induced a significant increase (*p* < 0.05) in mid-gestation hfBECs after 24 h exposure to the highest dose (1 μg/mL; Fig. 2H). In early gestation, overall ssRNA significantly increased in BCRP activity at 4 h (P < 0.02) and 24 h (P < 0.002). ssRNA treatment significantly (*p* < 0.05-P < 0.01) increased BCRP activity at 4 h (0.01 μg/mL) and 24 h (0.001–0.1 μg/mL; Fig. 2I, J). In mid-gestation hfBECs, overall ssRNA significantly increased BCRP activity at 4 h (P < 0.01) and 24 h (P < 0.002). At specific doses, ssRNA significantly (*p* < 0.05-P < 0.01) increased BCRP activity at both 4 h (0.01–0.1 μg/mL) and 24 h (0.001 & 0.1 μg/mL; Fig. 2K, L).

### Effects of bacterial and viral PAMPs on P-gp and BCRP protein levels in hfBECs

**Fig. 3:**
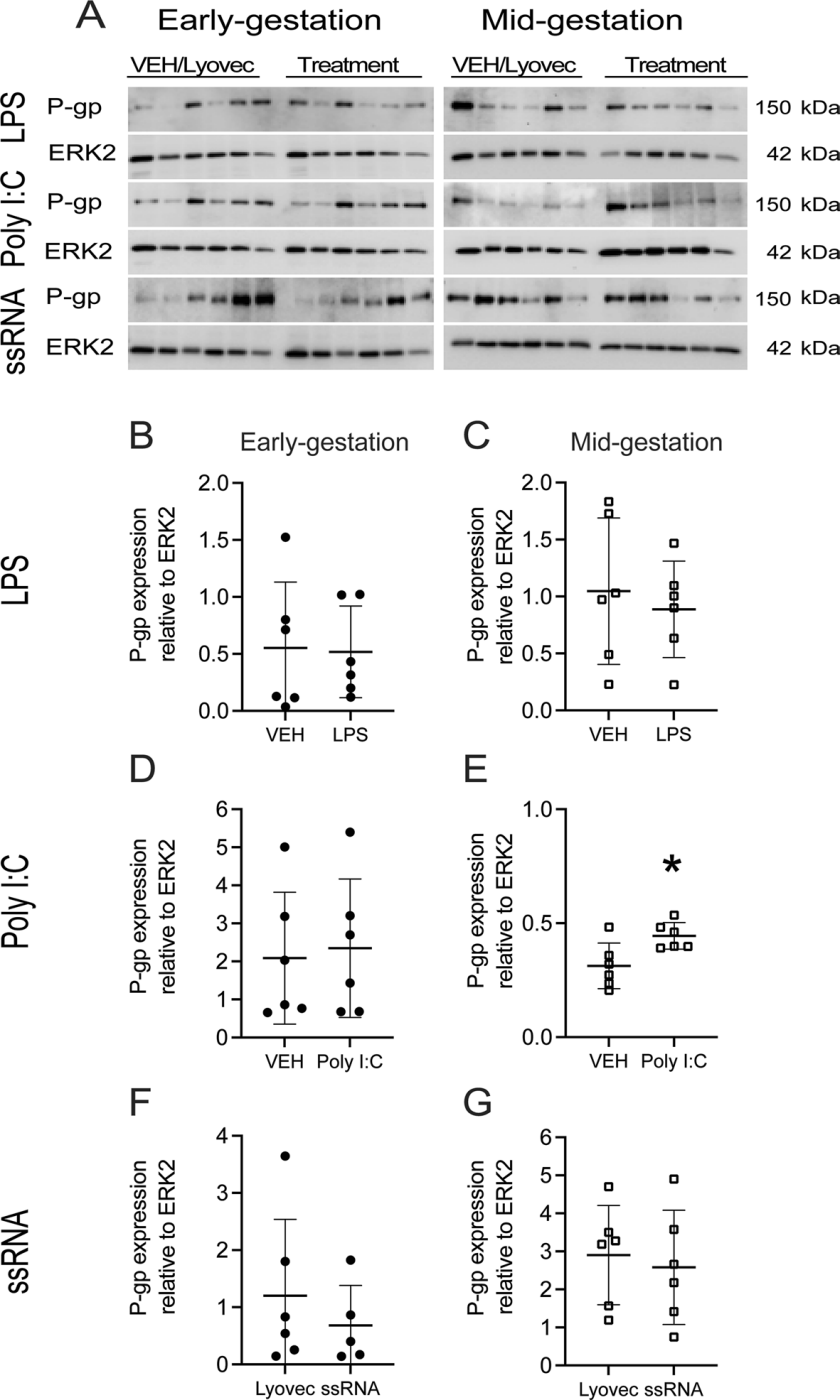
Poly I:C increased P-gp protein levels in second trimester human primary fetal brain endothelial cells (hfBECs). **A** Representative Western blot images and, **B–G** densitometric analysis of P-gp total protein levels, normalized to ERK2 (loading control for total protein), in early and mid-gestation hfBECs following treatment with LPS (0.01 µg/mL), Poly I:C (1 µg/mL), ssRNA (0.001 µg/mL) or vehicle for 24 h. Data are expressed as means ± SD. N = 6/group (if N < 6, an outlier has been removed). Statistical differences were tested using a paired *t*-test. *p < 0.05

**Fig. 4:**
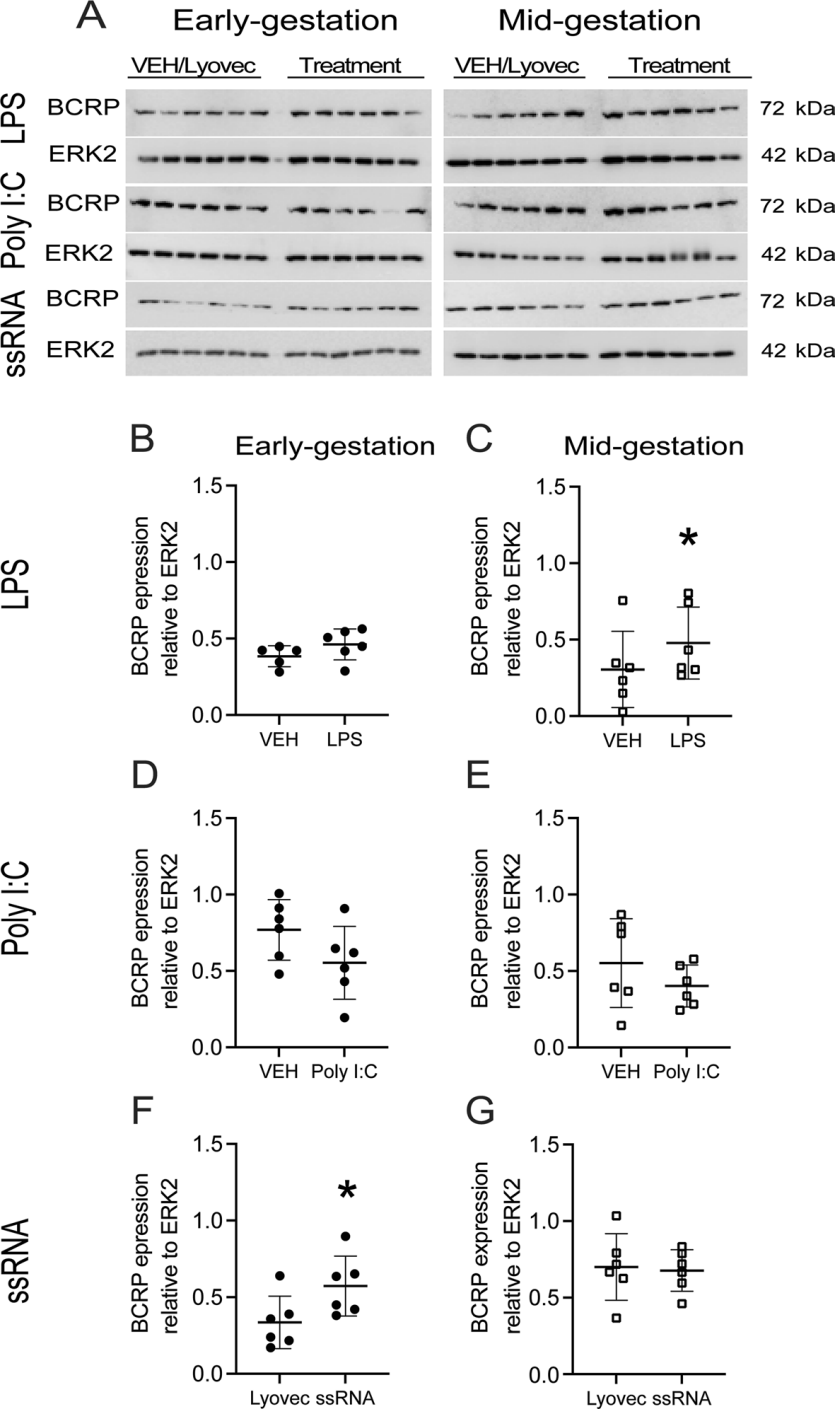
LPS and ssRNA increase BCRP protein levels in human primary fetal brain endothelial cells (hfBECs), in a gestational-age dependent manner. **A** Representative Western blot images and **B–G** densitometric analysis of BCRP total protein levels, normalized to ERK2 (loading control for total protein), in early and mid-gestation hfBECs following treatment with LPS (0.01 µg/mL), Poly I:C (1 µg/mL), ssRNA (0.001 µg/mL) or vehicle for 24 h. Data are expressed as means ± SD. N = 6/group. Statistical differences were tested using a paired *t*-test. *p < 0.05

In order to determine whether bacterial and viral PAMPs had an effect on P-gp and BCRP protein levels, hfBECs derived in early and mid-gestation were treated with LPS, Poly I:C and ssRNA for 24 h. LPS (0.01 µg/mL) and ssRNA (0.001 µg/mL) treatment had no effect on P-gp total protein level in early and mid-gestation hfBECs (Fig. 3A–D and F, G). Poly I:C (1 µg/mL) treatment induced a significant (*p* < 0.05) increase in P-gp protein in hfBECs derived in mid but not early-gestation (Fig. 3D, E). LPS treatment (Fig. 4A–C) induced a significant increase (*p* < 0.05) in BCRP protein in hfBECs from mid-gestation (Fig. 4C). Poly I:C treatment had no effect on BCRP protein levels (Fig. 4A, D and E), whereas ssRNA treatment significantly increased (*p* < 0.05) BCRP total protein levels but only in early-gestation hfBECs (Fig. 4F).

### Bacterial and viral PAMPs induce specific pro-inflammatory response but have limited effects on *ABCB1*/*ABCG2* levels in hfBECs

**Fig. 5:**
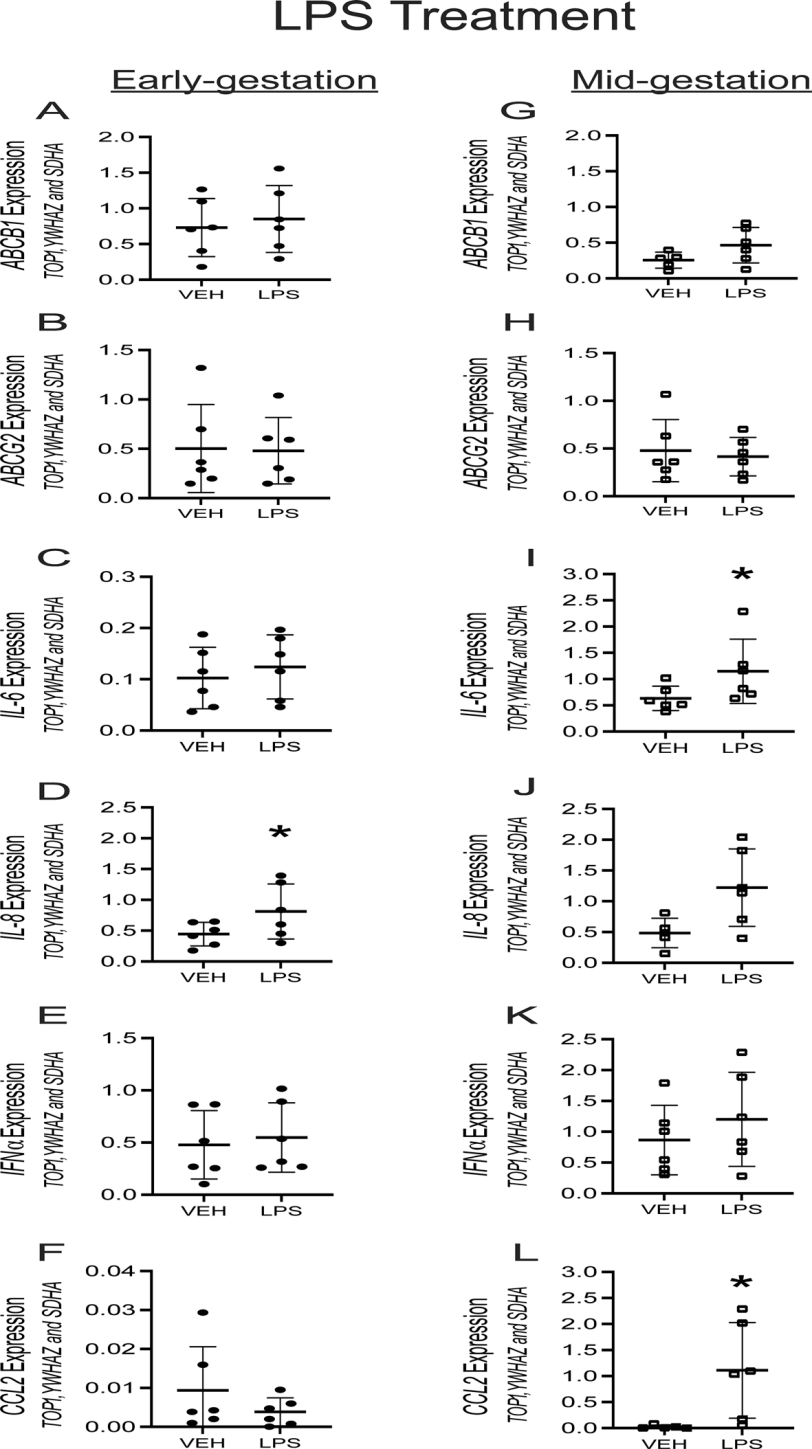
Effect of bacterial PAMP exposure (LPS 0.01 µg/mL or vehicle for 24 h) on total *ABCB1/ABCG2*, cytokines and chemokine mRNA levels in human primary fetal brain endothelial cells (hfBECs) derived in early and mid-gestation. Relative *ABCB1*/*ABCG2,* cytokines *(IL-6, IL-8, INFα)* and chemokine *(CCL2)* mRNA expression in early (**A–F**) and mid- (**G–L**) gestation hfBECs. Data are expressed as means ± SD. N = 6/group (if N < 6, an outlier has been removed). Statistical differences were tested using a paired *t*-test. *p < 0.05. mRNA values from LPS and Poly I:C (Fig. 6) treated groups were compared to the same vehicle controls (treated with water)

**Fig. 6:**
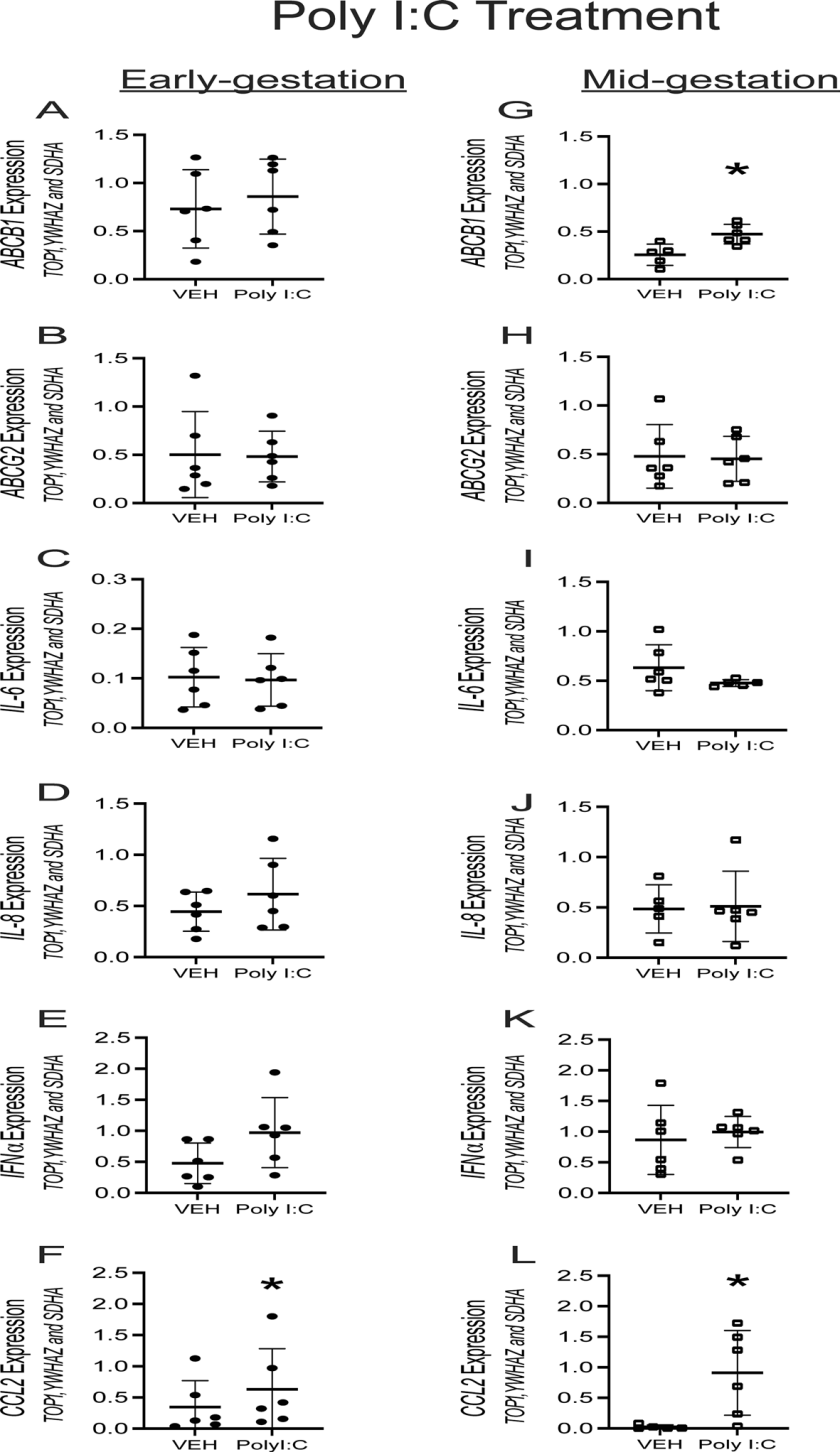
Effect of viral PAMP exposure (Poly I:C 1 µg/mL or vehicle for 24 h) on total *ABCB1/ABCG2*, cytokines and chemokine mRNA levels in human primary fetal brain endothelial cells (hfBECs) derived in early and mid-gestation. Relative *ABCB1*/*ABCG2,* cytokines *(IL-6, IL-8, INFα)* and chemokine *(CCL2)* mRNA expression in early (**A–F**) and mid- (**G–L**) gestation hfBECs. Data are expressed as means ± SD. N = 6/group (if N < 6, an outlier has been removed). Statistical differences were tested using a paired *t*-test. *p < 0.05. mRNA values from LPS (Fig. 5) and Poly I:C treated groups were compared to the same vehicle controls (treated with water), except for *CCL2* in early gestation (**F**) which was run in a separate PCR

In order to characterize the pro-inflammatory response of bacterial and viral PAMPs on hfBECs, cells were treated with LPS, Poly I:C and ssRNA for 24 h and the mRNA expression of *ABCB1* and *ABCG2*, as well as specific pro-inflammatory cytokines / chemokine were investigated. LPS (0.01 µg/mL) treatment did not affect *ABCB1* and *ABCG2* mRNA levels in early and mid-gestation hfBECs (Fig. 5A, B , G and H). Since cytokine and chemokine release modulates responses to bacterial and viral infection, we evaluated the induction of selected interleukin/chemokines, *IL-6, IL-8, IFNα* and *CCL2* mRNA expression following treatment of hfBECs with PAMPS. LPS treatment significantly increased the levels of *IL-6* and *CCL2* mRNA (*p* < 0.05; Fig. 5I and L) and promoted a trend for increased *IL-8* mRNA (*p* = 0.07; Fig. 5J) in mid-gestation. In early gestation, LPS increased the levels of *IL-8* mRNA (*p* < 0.05) (Fig. 5D), but had no effect on IL-6, IFN*α or CCL2* mRNA (Fig. 5C, E and F). Poly I:C (1 µg/mL) treatment induced a significant increase in *ABCB1* mRNA levels in hfBECs derived in mid but not early-gestation (Fig. 6A and G). Poly(I:C) treatment increased (*p* < 0.05) the expression of *CCL2* mRNA in early and mid-gestation but had no effect on *IL-6/8* and *INFα* mRNA expression (Fig. 6C–F and I–L). mRNA expression was not altered by ssRNA in either early or mid-gestation hfBECs for any of the genes assessed at 24 h following treatment (data not shown).

### Developmental expression of bacterial and viral sensing TLRs in early and mid-gestation hfBECs

**Fig. 7:**
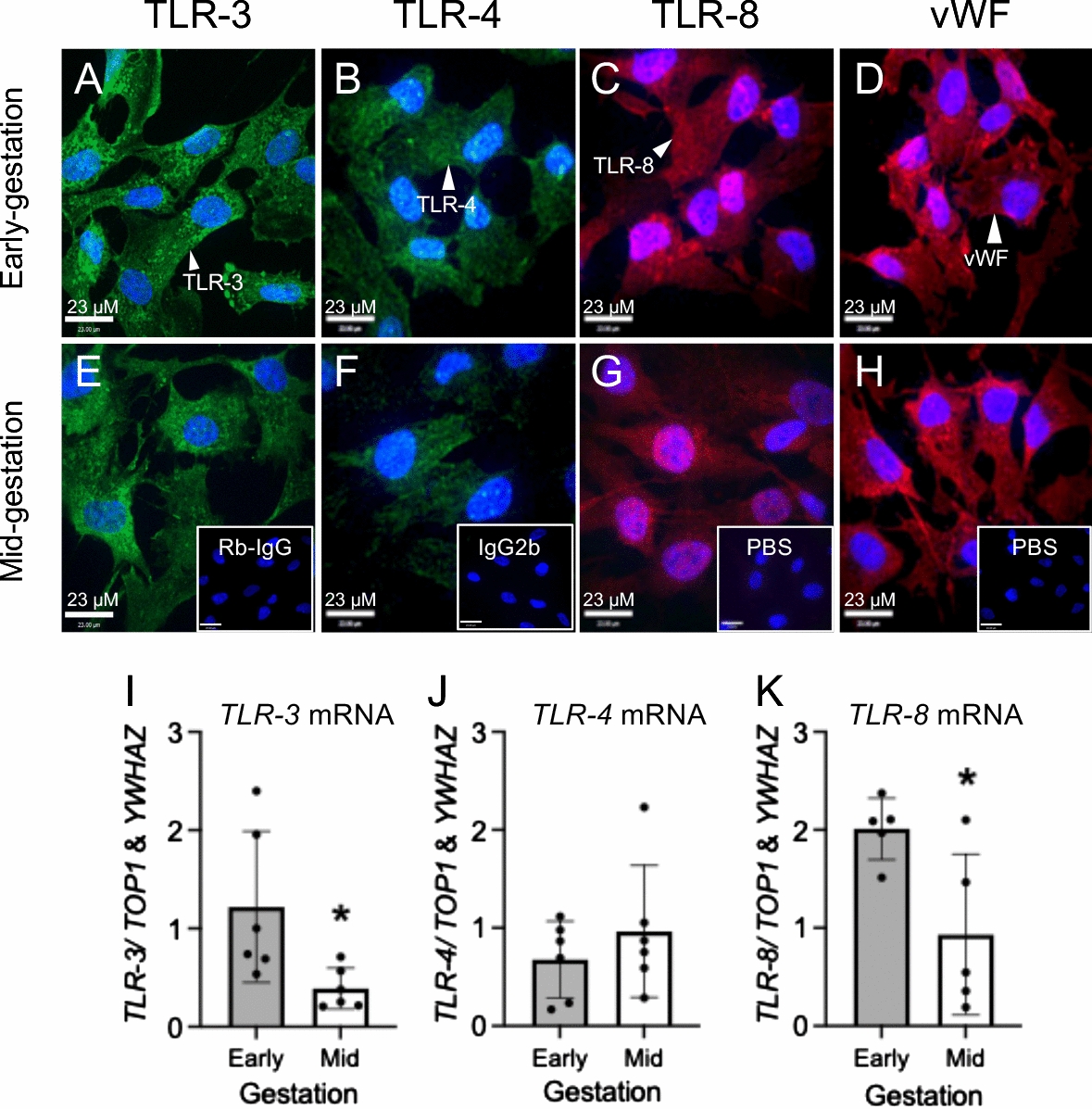
Expression and localization of bacterial and viral sensing toll like receptors (TLRs) in early and mid-gestation trimester isolated human fetal brain endothelial cells (hfBECs). Representative immunofluorescence photomicrographs of TLR-3 (green), TLR-4 (green) and TLR-8 (red) and von Willebrand Factor (vWF; endothelial cell marker (red)) in early (**A–D**) and mid-gestation (**E–H**) hfBECs. Blue indicates DAPI staining. Inserts show rabbit IgG (**E**), IgG2b (**F**) and PBS (**G**, **H**) negative controls. Scale bars = 23 μm. *TLR-3* (**I**), *TLR-4* (**J**) and *TLR-8* (**K**) mRNA levels in early and mid-gestation hfBECs. Early and mid-gestation hfBECs were cultured in the same batch to allow comparison between early and mid-gestation subjects. Data are expressed as means ± SD. N = 6/group (if N < 6, an outlier has been removed). Statistical analysis was conducted using an unpaired t-test. *p < 0.05

We next determined whether there are developmental differences in the mRNA expression of TLRs 3, 4 and 8. TLRs 3, 4 and 8 were localized to the cytoplasm of early and mid-gestation hfBECs, with similar patterns of cell localization (Fig. 7A–C and E–G). However, TLR-3 and TLR-8 also exhibited staining in intracellular vesicles contained within the cytoplasm of early and mid-gestation hfBECs (Fig. 7A, E and C, G). The majority of hfBECs were positive for the endothelial cell marker, vWF (Fig. 7D and H). *TLR-3, TLR-4* and *TLR-8* mRNA was detected in early and mid-gestation hfBECs, however, levels of *TLR-3* and *TLR-8* mRNA significantly decreased (*p* < 0.05) in mid compared to early-gestation (Fig. 7I, K). There were no differences in *TLR-4* mRNA levels between the two ages.

## Discussion

This is the first study to report the effect of bacterial and/or viral PAMPs on P-gp and / or BCRP expression and function in hfBECs derived in early and mid-gestation. This differential regulation may provide a means for the transporter function in the BBB to be maintained even during infection. These findings have important implications for the distribution of MDR substrates (various drugs, toxins, cytokines, chemokine, steroid hormones and waste products) within the CNS during prenatal bacterial and viral infections or inflammation. Further, the effects of the individual PAMPs differ from early to mid-gestation potentially indicating a developmentally-specific impact.

The bacterial PAMP, LPS, which activates TLR4 [43], decreased P-gp activity while inducing a significant increase in BCRP activity in both early and mid-gestation hfBECs. This is in contrast with previous findings from our group, where we detected a significant increase in P-gp activity and a decrease in BCRP function in adult hCMEC/D3 BECs exposed to LPS [11]. These divergent results may be attributed to the immortalized vs primary nature of the cells, to the different cytokine / chemokine responses or alternatively to the developmental stages (fetal x adults) of the BECs in the two studies. In addition, even though LPS impacted the activity of the transporters, it had a limited effect on P-gp or BCRP protein or mRNA levels, except for an increase in BCRP protein in mid-gestation hfBECs. The lack of association between transporter expression and activity raises the possibility that post-translational processing, such as phosphorylation or glycosylation [44–48], may be more important in determining P-gp and / or BCRP activity, and that these processes may be modulated by LPS (and Poly I:C and ssRNA, see below).

The dsRNA viral PAMP, Poly I:C, which activates TLR3 [49], decreased P-gp activity and increased BCRP activity in hfBECs, though the latter was limited to high dose treatment in mid-gestation. There was limited effect on the expression of the drug transporters. The decrease in P-gp activity is consistent with previous findings from our group where treatment with Poly I:C decreased P-gp activity in the mid-gestation fetal blood–brain barrier of the mouse, in vivo [13]. This suggests that TLR3-activation has the potential to increase fetal brain exposure to P-gp xenobiotic substrates in mid-pregnancy both in the human and in the mouse. However, Poly I:C produced opposite effects in adult (hCMEC/D3), where exposure to the viral mimetic resulted in a significant increase in P-gp activity while BCRP activity was decreased. This may represent an important developmental difference in the BBB response to viral infection when comparing hfBECs x hCMEC/D3, or alternatively, as previously discussed, these differences may be attributed to the immortalized x primary nature of the cells or the different cytokine / chemokine responses observed in the different studies. Future studies exposing primary adult brain endothelial cells to LPS, Poly I:C and ssRNA should be undertaken in order to confirm any developmental difference in brain endothelial cell response to PAMPs. Nevertheless, together, our data suggest that the effects of bacterial or viral PAMPs act similarly in hfBECs to decrease P-gp activity while increasing the activity of BCRP (mid-gestation only), though the impact that this differential regulation has on the integrity of the BBB remains to be determined.

ssRNA, the single-stranded RNA viral PAMP, which primarily activates TLR7/8 [50], decreased P-gp activity while increased BCRP activity in both early and mid-gestation and BCRP protein levels in early-gestation. Interestingly, ssRNA did not increase mRNA levels of specific cytokines and chemokine. These latter findings are consistent with results that we obtained following ssRNA treatment of hCMEC/D3 [11]. Interestingly, *Abcg2* mRNA expression in brain capillaries of adult *Abcb1a − /-* knockout mice is threefold higher compared to wild-type controls [51]; suggesting that a reduction in P-gp expression, may lead to a compensatory increase in *Abcg2* mRNA to maintain a level of protection at the BBB. Importantly, in the present study we identified that ssRNA, LPS and Poly I:C decreased P-gp while increasing BCRP activity. Together, these results indicate that a compensatory mechanism between BRCP and P-gp activity may exist in developing BECs. However, there appears to be some tissue-specificity in the impact of infection on BCRP, as we previously showed that ssRNA (and LPS) treatment of placental extravillous trophoblast cells (HTR-8/SVneo) decreased BCRP and *ABCG2* mRNA expression, though activity was not measured [52].

Fitzgerald et al. [53] reported that TLRs play an essential role in defending against pathogenic microbial infection through the induction of inflammatory cytokines and type I interferons, as well as mediating changes in the expression of key chemokines and pro-inflammatory cytokines in human epithelial cells [54]. Different pathogens related to poor pregnancy outcomes activate the TLRs 3, 4 and 7/8. Gram-negative bacteria *Escherichia coli*, *Ureaplasma urealyticum*, *Mycoplasma hominis* activate TLR-4 [25, 26]. In contrast, the TLR-3 is activated by viruses including acute respiratory syndrome coronavirus 2 (SARS-CoV-2), CMV, dengue virus (DENV), herpes simplex (HSV), VZV and ZIKV [11, 55–57]. While TLR-7/8 are activated by Chikungunya (CHIKV), DENV, human immunodeficiency virus (HIV), SARS-CoV-2 and ZIKV [11, 57–59].

The mechanisms by which bacterial and viral PAMPs modify expression of the MDR drug-transporters is not fully understood. However, these PAMPs bind to specific TLRs which are localized to hfBECs derived from both early and mid-gestation. Thus, the developmental profile of *TLR* mRNA expression and localization in early and mid-gestation hfBEC likely impacts the cellular response to bacteria and viruses, though specific mechanisms underlying these responses require further investigation. Higher mRNA levels of the viral sensors TLR-3 and 8 were found in early-gestation compared to mid-gestation, demonstrating a gestational-age-dependent mRNA expression at the developing BBB, however how these findings translate to the capacity of these cells to respond to specific infective challenges, require further investigation. Nevertheless, this finding is consistent with the increased brain sensitivity to viral infection in earlier stages of pregnancy [60]. A previous study from an independent group reported that the TLR-7 ligand imiquimod did not induce marked immune activation in immortalized SV-40 human cerebral microvascular endothelial cells (hCMVECs) [61], As such, we focused on the protein localization and mRNA levels of TLR-8 in isolated hfBECs.

In the current study, LPS and poly I:C treatments induced specific pro-inflammatory responses which varied according to gestational age. LPS increased mRNA levels of *IL-8* (early) and *IL-6* (mid-gestation) as well as *CCL2* (mid-gestation). Poly I:C increased mRNA levels of *CCL2* in early and mid-gestation in hfBECs. LPS has been demonstrated to increase IL-6, IL-8 and CCL2 in vivo and in vitro, at the level of mRNA and protein in various cells and tissues including adult peripheral blood [18, 62], hCMEC/D3 BECs [11] and placenta [35, 37, 63]. Poly I:C exposure has also been shown to increase peripheral blood levels of IL-6 and CCL2 in mice, in vivo [13, 16]. Surprisingly, ssRNA did not elicit changes in cytokine / chemokine expression in early and mid-gestation hfBECs. Previously, we demonstrated that ssRNA challenge did not alter *IL-6* mRNA levels in adult hCMEC/D3 BECs [11]. Accordingly, the TLR-7 ligand, imiquimod, did not induce alterations in the mRNA levels of *IL-6*, *IL-8* and *CCL2* in hCMVECs [61], showing that TLR-7/8 immune activation is complex and does not involve induction of IL-6, IL-8 and CCL2 mRNA levels in developing and adult BECs, at least at the time points investigated in these studies. However, ssRNA may induce the activation of other unexplored pro-inflammatory mediators. Furthermore, we observed a non-linear, dose-dependent effect of LPS, poly I:C and ssRNA on P-gp and BCRP activity in hfBECs derived in early and mid-gestation. Although the specific mechanisms underlying this response are unknown, similar non-linear dose responses have been reported in adult hCMEC/D3 BECs following exposure to infective PAMPs [11]. One potential mechanism underlying this non-linear response, is the differential effect of PAMPs on the production of specific cytokine and chemokines that has previously been reported in adult hCMEC/D3 BECs [11]. In this connection, we have previously demonstrated an age and dose-dependent effect of cytokines (IL-1β, IL-6 or TNF-α) on P-gp activity in BECs derived from the guinea pig at various stages of development [12]. In addition, adult porcine brain capillary endothelial cells exhibited a complex and robust P-gp/*ABCB1* and BCRP/*ABCG2* response (expression and or function) following IL-1β and TNF-α exposure [64]. Therefore, it is possible that local (and specific) cytokine / chemokine release elicited by bacterial and viral exposure may have modulatory effects that are additive to the direct actions of TLR activation, controlling P-gp and BCRP activity in developing BECs; a hypothesis that requires further investigation. Notwithstanding, together these data show that bacterial and viral infection may target TLR proteins located at BECs as early as the first trimester of pregnancy and stimulate the expression of specific pro-inflammatory mediators in the developing BBB.

The present study raises the possibility that bacterial and viral infection through activation of TLR-3, TLR-4 and TLR-7/8, have the potential to alter fetal brain protection. In this context, decreased P-gp activity in hfBECs elicited by LPS, Poly I:C and ssRNA may increase brain accumulation of P-gp substrates relevant to neurodevelopment such as cytokines and chemokines (e.g. IL-1β, IL-2, IL-4, IL-6, IFNγ, tumor necrosis factor [TNF] and CCL2), endogenous and synthetic steroid hormones (e.g. cortisol, testosterone, betamethasone), xenobiotics (e.g. antibiotics, antidepressants, antivirals, environmental toxins) and waste products (bilirubin), while likely promoting concomitant exclusion of BCRP substrates such as folate (essential for normal neurodevelopment) and sphingolipids from the brain (important for myelin stability) [7, 65–67]. Altered brain distribution of such substrates in cases of in utero infection may contribute to white matter injury commonly associated with intrauterine bacterial or polymicrobial infection (chorioamnionitis), since increases in cytokine levels in the developing brain may induce activation of microglia and astrocytes leading to fetal brain neuroinflammation and white matter disruption [27]. This is particularly important since levels of endogenous P-gp substrates in the fetal circulation may already be high during infection. We have previously shown that LPS inhibits placental P-gp activity and increases the accumulation of P-gp substrates in the fetal peripheral circulation [62]. Furthermore, in hfBECs, P-gp protein and *ABCB1* mRNA as well as BCRP protein levels decrease from early to mid-gestation [9]. Other studies have shown that P-gp immunostaining in the adult human brain is higher than in the mid and late-gestation fetal brain [68]. Together, these studies would suggest that expression of MDR transporters is developmentally regulated in the fetal BBB. In the present study, we showed that LPS, poly I:C and ssRNA exposure increased P-gp and BCRP protein levels in a PAMP-specific manner. As such, it is possible that bacterial and viral infections may disrupt the expression pattern of MDR transporters in the developing BBB, and this may have long-term consequences on brain development.

It is recognized that the relatively low number of subjects represents a limitation in the present study. However, availability of well characterized human fetal brain specimens, particularly for in vitro research is highly limited. Further, we have very carefully characterized the cells used in the present study [9]. It is also important to note that a sample size of six per group or less has been previously utilized in human [9, 19, 35, 37, 39, 52, 69] and animal [13, 21, 40, 62] studies of drug transporter expression and function during development. In addition, as mandated by our REB, we are unable to collect any clinical information on the fetal tissues collected. Clinical differences such as maternal age, parity, BMI and ethnicity, as well as fetal sex could account for the within group variability found in some of our results. Another limitation of this type of study is the difficulty in directly relating the dose of PAMPs used in in vitro studies to potential in vivo exposure of the fetal brain during infection. These points should be taken into consideration when interpreting our findings. Notwithstanding, our results are novel and show that hfBECs express bacterial and viral TLRs capable of recognizing and responding to bacterial and viral infections, which have the potential to disrupt function of the BBB as early as the first trimester of pregnancy.

## Conclusions

Exposure to the bacterial and viral PAMPs, LPS, Poly I:C and ssRNA resulted in a decrease in P-gp activity while concomitantly increasing the activity of BCRP in hfBECs. Since these PAMPs differentially impact the activity of these two drug-transporters in a time and dose-dependent manner, it would likely lead to perturbations in the efficacy of drug / toxin efflux across the developing BBB. Given the susceptibility of the developing brain to these agents, further research is needed to fully understand the contribution of the drug-transporters in protecting the developing brain from cytokines, toxins and chemicals, as well as modulating access of endogenous compounds and therapeutic agents into the developing brain. Reduced P-gp activity in the developing BBB has the potential to increase fetal brain accumulation of P-gp substrates with potential neurotoxic effects such as amyloid β-peptide, bilirubin, endogenous and synthetic glucocorticoids, endosulfan (an organochlorine insecticide), and specific cytokines/chemokines [4, 7, 70–75]. Whereas, increased BCRP activity in the developing BBB, induced by infection, has the potential to lower the brain levels of specific BCRP substrates relevant to brain development including folate and sphingolipids as well as xenobiotics [7, 76, 77]. Future studies should investigate the pharmacokinetics of specific P-gp and BCRP substrates of interest in the developing CNS in cases of maternal infection.

## Supplementary Information

**Additional file 1: Figure. S1.** Effect of bacterial and viral PAMPs exposure on esterase activity in early (n=5) and mid-gestation (n=6) human primary fetal brain endothelial cells (hfBECs). Relative fluorescence units (RFU) in lysed hfBECs following treatment with 0.01 µg/mL LPS and Poly (I:C) (A-B) or 0.001 ug/mL ssRNA (C-D) compared to control (respective vehicles) for 24h. RFU is displayed as mean ± SD. LPS and Poly I:C data were analyzed by One-way ANOVA against the control (vehicle) group. ssRNA data were analysed using a paired *t*-test.

## Data Availability

All data generated or analyzed during this study are included in this published article and its supplementary information files.

## Abbreviations

ABC: ATP-binding cassette
BCRP: Breast cancer resistance protein
Ca-AM: Calcein-acetoxymethyl ester
CCL2: C–C motif chemokine ligand 2
Ce6: Chlorin e6
CHIKV: Chikungunya
CMV: Cytomegalovirus
CNS: Central nervous system
(CS)-FBS: Charcoal-stripped fetal bovine serum
DENV: Dengue virus
DMEM: Dulbecco’s Modified Eagle Medium
dsRNA: Double-stranded viral RNA
hCMEC/D3: Human cerebral microvascular endothelial
hCMVECs: SV40 human cerebral microvascular endothelial cells
hfBECs: Human fetal brain endothelial cells
HIV: Human immunodeficiency virus
HSV: Herpes simplex
IFN: Interferon
IL: Interleukin
LPS: Lipopolysaccharide
MCP-1: Monocyte chemoattractant protein 1
MDR: Multidrug resistance
PBCEC: Porcine brain capillary endothelial cells
P-gp: P-glycoprotein
PVDF: Polyvinylidene fluoride
Poly I:C: Polyinosinic polycytidylic acid
SARS-CoV-2: Acute respiratory syndrome coronavirus 2
SDHA: Succinate ubiquinone oxidoreductase
ssRNA: Single-stranded RNA
SV40: Simian virus 40 large T antigen
TLR: Toll like receptor
TNF: Tumor necrosis factor
TOP1: DNA topoisomerase 1
VZV: Varicella-zoster virus
vWF: Von Willebrand factor
YWHAZ: The zeta polypeptide
ZIKV: Zika virus
