# Supplementary figures and images for: Effects of bacterial and viral pathogen-associated molecular patterns (PAMPs) on multidrug resistance (MDR) transporters in brain endothelial cells of the developing human blood–brain barrier
Source: Fluids Barriers CNS. 2023 Jan 31;20:8. doi: 10.1186/s12987-023-00409-4 (PMC9887585; doi:10.1186/s12987-023-00409-4)

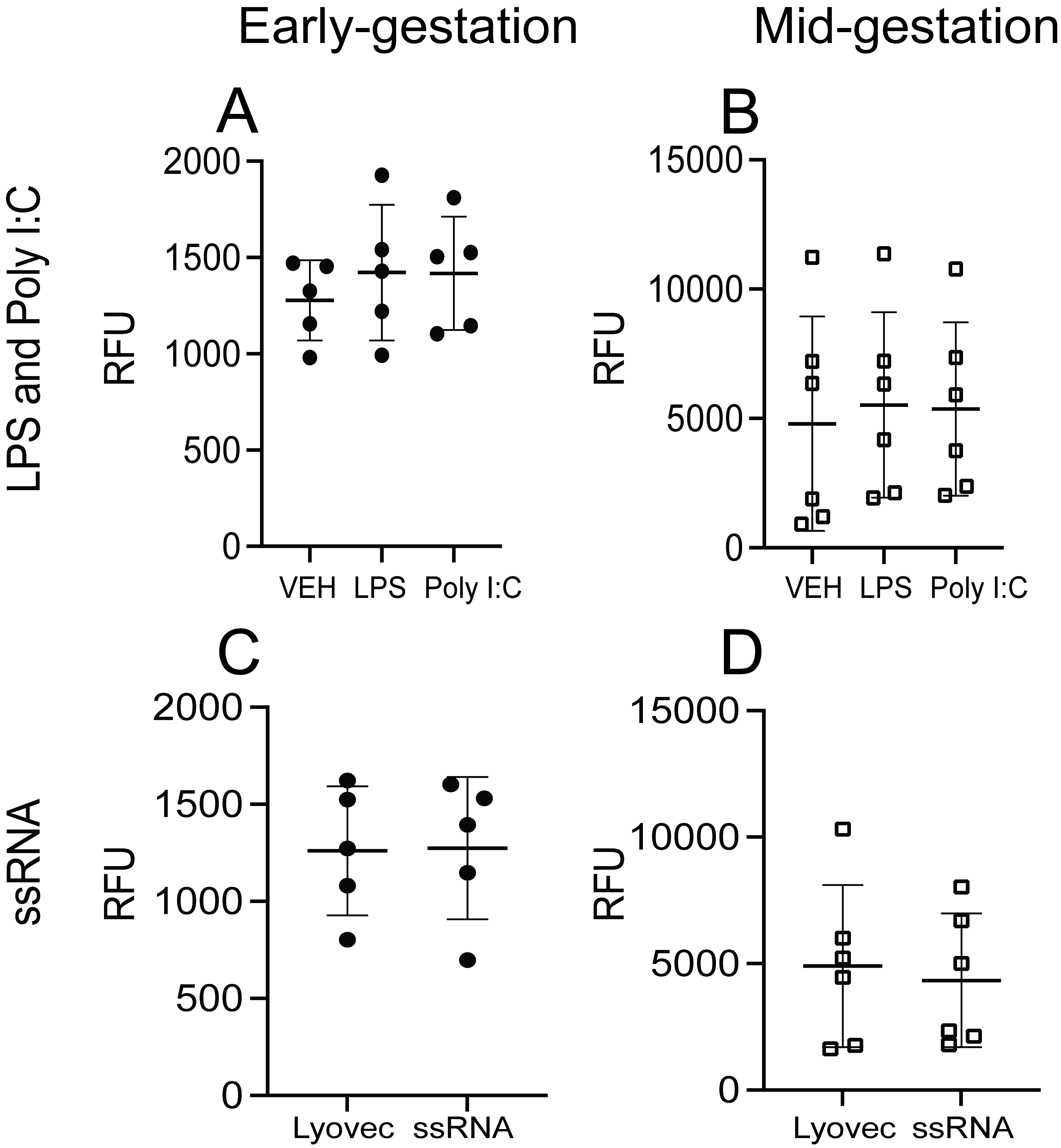

Supplement: Supplementary file 1 — Additional file 1: Figure. S1. Effect of bacterial and viral PAMPs exposure on esterase activity in early (n=5) and mid-gestation (n=6) human primary fetal brain endothelial cells (hfBECs). Relative fluorescence units (RFU) in lysed hfBECs following treatment with 0.01 µg/mL LPS and Poly (I:C) (A-B) or 0.001 ug/mL ssRNA (C-D) compared to control (respective vehicles) for 24h. RFU is displayed as mean ± SD. LPS and Poly I:C data were analyzed by One-way ANOVA against the control (vehicle) group. ssRNA data were analysed using a paired t-test. [file 12987_2023_409_MOESM1_ESM.tif]
